# Endogenous Network Modeling Reveals Mechanisms of Repair Schwann Cell Decline and Potential Recovery Targets

**DOI:** 10.3390/biology15131079

**Published:** 2026-07-06

**Authors:** Zongyi Zhou, Ruiqi Xiong, Shunlian Fu, Yang Su, Qiang Ao, Yong-Cong Chen, Ping Ao

**Affiliations:** 1 College of Biomedical Engineering, Sichuan University, Chengdu 610065, China; zhouzongyi@stu.scu.edu.cn (Z.Z.); ruiqixiong@shu.edu.cn (R.X.); 2 School of Clinical Medicine, Chengdu University of Traditional Chinese Medicine, Chengdu 610072, China; fushunlian@stu.cdutcm.edu.cn; 3 Shanghai Center for Quantitative Life Sciences & Department of Physics, Shanghai University, Shanghai 200444, China; suyang@shu.edu.cn; 4 National Engineering Research Center for Biomaterials, Sichuan University, Chengdu 610064, China; aoqiang@scu.edu.cn; 5 Shenzhen International Quantum Academy (IQASZ), Shenzhen 518048, China

**Keywords:** Schwann cell, peripheral nerve injury, nerve regeneration, chronic denervation, endogenous network, cell fate transition, repair Schwann cell

## Abstract

Schwann cells, the principal glial cells of the peripheral nervous system, play a central role in nerve repair following injury. Upon injury, mature Schwann cells dedifferentiate into repair Schwann cells. These processes are governed by complex gene regulatory networks, yet the quantitative dynamics of these processes remain unclear. Here, using a bottom-up systems biology approach, we constructed an endogenous regulatory network model based on experimentally validated interactions, without relying on high-throughput data as input. The model captures Schwann cell dedifferentiation dynamics and reveals a potential landscape composed of stable states and intermediate transition states. Simulations recapitulate post-injury trajectories and confirm the role of c-Jun upregulation in maintaining repair capacity. Furthermore, the model predicts multiple potential therapeutic targets, including tumor protein p53 (P53), c-Jun N-terminal kinase (JNK), and phosphatase and tensin homolog (PTEN), for sustaining repair competence. We also identify intrinsic heterogeneity within repair Schwann cells. Furthermore, we uncover key transition states that simultaneously connect repair-competent cells to both repair-deficient and apoptotic phenotypes. These intermediate states may represent critical regulatory bottlenecks and serve as key cellular targets for improving peripheral nerve regeneration. Overall, this work provides new insights into the precise regulation of Schwann cell fate and establishes a theoretical framework for regenerative medicine and clinical strategies in peripheral nerve repair.

## Simple Summary

Successful peripheral nerve regeneration depends on repair Schwann cells, which support damaged nerves after injury. However, during prolonged denervation these cells gradually lose their regenerative ability, leading to poor recovery outcomes. The mechanisms responsible for this process remain incompletely understood. In this study, we constructed a systems-level regulatory model of Schwann cells and used it to explore how different cellular states arise and change over time. The model identified healthy repair cells, dysfunctional repair cells, and apoptotic cells, together with the transition pathways connecting them. Importantly, our results suggest that repair Schwann cells are intrinsically heterogeneous, with some subpopulations showing a greater tendency to undergo degeneration and cell death than others. This finding provides a possible explanation for the progressive loss of Schwann cells observed after long-term nerve injury. The model predictions aligned with experimental evidence, transcriptomic datasets, and single-cell gene expression data. By revealing how repair Schwann cells decline and identifying potential molecular targets associated with this process, this work offers new insights into the biological mechanisms underlying failed nerve regeneration and may support the development of future strategies to improve peripheral nerve repair.

## 1. Introduction

Upon peripheral nerve injury, mature Schwann cells dedifferentiate into a transient repair phenotype that actively supports axonal regeneration [1,2]. The remarkable regenerative capacity of peripheral nerves is largely attributed to this plasticity of Schwann cells. Following injury, Schwann cells undergo a phenotypic transformation into a specialized reparative state, characterized by the downregulation of myelin-associated genes and the upregulation of pro-regenerative genes, including those encoding neurotrophic factors and cytokines involved in immune modulation. Concurrently, Schwann cells initiate myelin autophagy and recruit macrophages to facilitate the efficient clearance of myelin debris. They also reorganize into Büngner bands, which provide structural guidance for regenerating axons. Upon completion of regeneration, repair Schwann cells redifferentiate into either mature myelinating Schwann cells or non-myelinating Schwann cells, thereby restoring the functional architecture of the nerve [3,4].

Despite these reparative mechanisms, functional recovery following peripheral nerve injury remains suboptimal, a limitation largely driven by the inherently slow axonal regrowth and the transient reparative phenotype of Schwann cells. Over time, repair Schwann cells progressively lose their regenerative capacity, characterized by the downregulation of repair-associated genes and reduced expression of neurotrophic factors, which contribute to the failure of nerve regeneration [4,5,6]. Addressing chronic denervation of Schwann cells should be considered not only a therapeutic target but also a central objective of any effective strategy aimed at improving nerve regeneration. How to modulate these specialized Schwann cells to boost their reparative capacity and prevent functional decline over the protracted axonal regeneration period remains a critical research focus [4,7,8].

In recent years, numerous studies have revealed the critical roles of various transcription factors and signaling pathways in Schwann cell responses to injury. Previous research has systematically delineated the major transcriptional regulatory networks involved in Schwann cell myelination, including the roles of key factors such as SRY (sex determining region Y)-box transcription factor 10 (Sox10), POU class 3 homeobox 1 (Oct6/POU3F1) and POU class 3 homeobox 2 (Brn2/POU3F2), and early growth response 2 (EGR2/Krox20) in activating myelin-associated genes [9]. In addition, studies have shown that sustained expression of Sox2 in mice can inhibit myelination while inducing a proliferative, undifferentiated state [10]. In signal transducer and activator of transcription 3 (Stat3) knockout experiments in mice, researchers observed a significant reduction in Schwann cells following denervation, indicating that this gene is essential for the long-term maintenance of Schwann cell phenotypes [11]. Similarly, c-Jun knockout experiments demonstrated that loss of c-Jun led to substantial neuronal cell loss, impaired axonal regeneration, and reduced secretion of multiple neurotrophic factors. Jun proto-oncogene, AP-1 transcription factor subunit (c-Jun) has also been identified as a regulator of glial cell line derived neurotrophic factor (GDNF) and brain derived neurotrophic factor (BDNF) [12]. Collectively, these studies highlight the presence of numerous key transcription factors involved in Schwann cell regulation. Such traditional approaches are largely based on a linear regulatory paradigm, focusing on the role of individual genes or single signaling pathways in specific biological processes. As a result, they primarily explain the function and importance of these factors in nerve regeneration in an isolated manner.

However, such locally focused approaches are insufficient to fully elucidate system-level regulatory mechanisms. As illustrated by the “radio” analogy proposed by Lazebnik, biologists often demonstrate that the loss of a specific factor causes the system to “fall silent,” thereby identifying it as a key target and assuming that modulating this single component may be sufficient to “repair” the system [13]. Similarly, Davidson pointed out that observations at the level of individual genes cannot reveal the overall organizational structure of biological systems or the mechanisms underlying organ development, but instead provide only a microscopic and inherently limited perspective [14]. In the context of chronic denervation–induced systemic functional decline, the loss of repair capacity in Schwann cells is not determined by any single factor alone, but rather arises from dysregulated coordination among key molecules and signaling pathways within the regulatory network. Therefore, it is necessary to analyze these biological phenomena from a systems-level perspective.

In recent years, with the rapid development of high-throughput sequencing technologies, researchers have begun to systematically analyze gene expression profiles of Schwann cells in different states using transcriptome sequencing and single-cell RNA sequencing, thereby constructing their potential gene regulatory networks [15,16]. Using high-resolution omics, investigators analyzed human Schwann cells, fibroblasts, and neural tissues at both the protein and transcript levels, revealing that cultured human Schwann cells retain their inherent reparative capacity [17]. Although these studies have characterized the molecular features of Schwann cells during nerve repair at a systems level and provided valuable references for further elucidation of their regulatory mechanisms, such high-throughput data mainly provide a descriptive characterization of system states and are limited in revealing the underlying mechanisms and causal relationships driving these changes.

The fate determination and functional remodeling of Schwann cells depend on complex interactions among multiple key molecules and signaling pathways. Correlation-based analyses alone are insufficient to elucidate their underlying regulatory logic and dynamic evolution. It is therefore necessary to introduce research frameworks grounded in cell biology and evolutionary biology that can capture causal relationships and system-level dynamics. Such approaches allow for understanding the interaction mechanisms among these critical regulatory factors at the network level and further reveal the intrinsic drivers of Schwann cell state transitions and changes in their repair capacity [18,19].

Indeed, Schwann cell dedifferentiation is governed by a highly interconnected regulatory network involving extensive cross-talk among transcription factors and signaling pathways, which cannot be captured by simplified models, highlighting the need for a system-level network framework [8]. Conceptual foundations for such approaches trace back to the epigenetic and fitness landscapes proposed by Waddington and Wright, which provided topological representations of biological dynamics [20,21]. Kauffman later demonstrated that gene regulatory networks inherently converge to stable attractor states using stochastic Boolean models [22]. Building on these ideas, the first quantitative landscape was constructed for the bacteriophage lambda gene switch [23]. This work demonstrated that cellular state transitions could be quantitatively described within a landscape framework and provided a foundation for extending landscape-based approaches to more complex biological systems. In 2008, Ao and Hood et al. proposed an endogenous network model based on non-linear stochastic dynamical systems. The model describes molecular regulatory mechanisms of complex biological systems within the framework of systems biology [24]. This theory suggests that, during biological evolution, the molecules and cytokines within an organism collectively form a nonlinear and stochastic endogenous molecular–cellular network at the cellular level. The components of this network include tumor suppressor genes, oncogenes, and associated growth factors. This endogenous network can be quantitatively represented by the expression levels of key endogenous factors, forming a high-dimensional stochastic dynamical system. The nonlinear dynamic interactions among network components can generate multiple local steady states with either distinct or indistinct biological functions, which may correspond to specific cellular states observed experimentally. The endogenous network can autonomously form and sustain multiple steady states that persist over extended periods of time. These steady states collectively constitute the potential landscape, and the transitions of cellular states during differentiation can be interpreted as stochastic shifts between distinct steady states within this landscape. Compared with the prevalent linear additive thinking in traditional research, the global dynamic characteristics of the network provide a more comprehensive framework for understanding the mechanisms underlying disease onset and progression [25]. This framework has since been successfully applied to multiple cancers and to developmental patterning of the telencephalon [25,26,27].

Building on existing experimental evidence, we constructed an endogenous core regulatory network of Schwann cells in the context of peripheral nerve injury from a systems-level perspective. Dynamic modeling of this regulatory network revealed multiple steady states and transitional states, which are interconnected to form a dedifferentiation landscape that captures the topological structure of Schwann cell state transitions and elucidates the molecular processes underlying chronic denervation. Furthermore, we simulated targeted interventions within the network and successfully recapitulated experimentally validated targets required for maintaining the reparative activity of Schwann cells, while also predicting several additional candidate therapeutic targets.

## 2. Materials and Methods

### 2.1. Construction and Analysis of the Endogenous Molecular-Cellular Network

To construct an endogenous network for a specific research object, the first step is to select modules and pathways according to biological phenomena and functions. These modules are both integral components of, and relatively independent from, the entire biological system. Interactions occur among key endogenous factors within each module, while higher-level functions, such as cell fate determination, can be achieved through crosstalk between modules. This hierarchical organization resembles the principle of modular organization [28]. Based on experimental evidence reported in the literature, key endogenous factors involved in these modules were subsequently selected, and their regulatory relationships were curated to establish the endogenous network. The integrated interactions among these factors collectively defined the network architecture. The resulting network was then subjected to dynamical analyses using both Boolean dynamics and ordinary differential equation (ODE)-based approaches. To evaluate the biological relevance and reliability of the model, the computational results were compared with available low-throughput and high-throughput experimental data. Through iterative refinement and validation, the network was progressively improved to generate experimentally testable predictions and to computationally predict potential therapeutic targets that may contribute to maintaining the reparative phenotype of Schwann cells following peripheral nerve injury.

In the present study, the endogenous network was constructed based on the sequential responses of Schwann cells to peripheral nerve injury. Accordingly, four key functional modules and five major signaling pathways were incorporated into the network, including the Schwann cell differentiation, inflammation, proliferation, and apoptosis modules, as well as the JNK, AKT serine/threonine kinase (Akt), Ras signaling pathway (Ras), extracellular signal-regulated kinase (ERK), and PTEN pathways. Detailed information on all modules, their constituent factors, and their functional roles is provided in Table S1 (Part A, Supplementary Materials). It should be noted that factors were assigned to functional modules according to their predominant regulatory roles and network connectivity within the endogenous regulatory network. This classification was intended to reflect the organizational structure of the network rather than exclusive functional specificity, as extensive crosstalk exists among different modules. Accordingly, multifunctional regulators may contribute to multiple biological processes and maintain regulatory interactions across different modules despite being assigned to a specific functional category. The criteria used for the selection of modules and core factors are described below.

### 2.2. Selection Criteria for Modules and Core Factors in the Endogenous Network

Certain cellular functions, such as signal transduction, are carried out by functional modules within the cell. These modules consist of interacting molecules that regulate each other, and integrating insights from biological phenomena with molecular-level studies can facilitate a deeper understanding of their underlying mechanisms [28]. The construction of endogenous networks is grounded in biological phenomena [24]. First, we select modules based on observed cellular or tissue-level phenomena and their associated functions. Following peripheral nerve injury, Schwann cells undergo significant morphological and functional changes. According to current experimental evidence, they dedifferentiate from myelinating and non-myelinating Schwann cells into a flattened repair phenotype. Accordingly, a Schwann cell differentiation module was incorporated into the network [29].

During Wallerian degeneration, Schwann cells release pro-inflammatory cytokines and recruit macrophages to cooperatively clear myelin debris, thereby creating a favorable environment for nerve regeneration. Based on this process, an inflammation module was included [3,30].

In response to injury, Schwann cells exit the quiescent state and re-enter the cell cycle, leading to proliferation that fills the injured area and contributes to the formation of Bands of Büngner. Therefore, a cell proliferation module was added [31,32,33].

Due to the slow rate of axonal growth, nerve repair often requires an extended period. Under long-term denervation, Schwann cells are unable to maintain the repair phenotype, accompanied by downregulation of repair-associated factors and even apoptosis. Accordingly, an apoptosis module was incorporated into the network [4,34].

Finally, during the transmission of dedifferentiation signals in Schwann cells, several key signaling pathways play critical roles, including the JNK, ERK, and Akt pathways. These pathways are involved in multiple processes of Schwann cell dedifferentiation and were therefore integrated into the network as essential signaling components [8,35]. The selection criteria for core factors are described below.

The regulatory network constructed in this study does not attempt to encompass all molecular components at the genome-wide level. This reflects the current state of biological knowledge, as many regulatory factors involved in Schwann cell repair remain incompletely characterized, and even among known factors, most molecular interactions and intracellular kinetic parameters are not fully defined [36,37,38]. For example, yes-associated protein 1 (YAP) and WW domain containing transcription regulator 1 (TAZ) have been reported to contribute to Schwann cell myelination [39]; however, this functional role is largely captured in our network by the core myelination-associated regulators Krox20 and Sox10. Therefore, the effects of YAP/TAZ can be incorporated into these core factors. In addition, the role of YAP/TAZ in promoting Schwann cell proliferation after injury remains context-dependent and has been reported inconsistently across studies [40,41]. Introducing YAP/TAZ as independent nodes may thus introduce redundancy and additional uncertainty, without substantially improving the functional representation of the myelination program. Similarly, transcription factor EB/3 (TFEB/3) have been implicated in Schwann cell dedifferentiation and injury responses, but these functions are largely represented in the current model by key regulators such as c-Jun and notch receptor 1 (Notch). Moreover, the currently available data on TFEB/3-mediated regulatory interactions remain relatively limited, making it difficult to define their precise roles within a mechanistic network framework. Collectively, the network was restricted to well-characterized core regulators with clearly established regulatory functions, ensuring both biological credibility and interpretability.

Biological systems are inherently organized in a hierarchical manner [42,43,44]. Consistently, single-gene knockout experiments have shown that most genes exert limited phenotypic effects when disrupted, supporting the existence of a core regulatory architecture [45]. In this context, upstream master regulators such as c-Jun and Krox20 were selected to represent major functional programs. These factors were selected based on consistent experimental evidence supporting their central regulatory roles. Using Affymetrix whole-genome microarray analysis of injured mouse nerves, Arthur-Farraj et al. demonstrated that c-Jun is a key regulator of the Schwann cell injury response, controlling the expression of trophic factors and adhesion molecules, and contributing to regeneration track formation and myelin clearance [46]. Topilko et al. generated a Krox20 null allele (Krox20−/−) mouse model and demonstrated that loss of Krox20 arrests Schwann cells at an early differentiation stage, abolishes the expression of key myelin genes such as MBP and P0, and ultimately prevents peripheral nerve myelination [47]. In Runx2 knockout mouse models, researchers have observed impaired Schwann cell proliferation, migration, and axonal regeneration, indicating that Runx2 plays an important role in Schwann cell migration and peripheral nerve repair [48]. As a master regulator of dedifferentiation, c-Jun can also activate downstream effectors to promote Schwann cell dedifferentiation, migration, and proliferation [12,46]. Therefore, we integrated the functional role of Runx2 into that of c-Jun in our model.

In the present network, these core nodes are assumed to integrate the aggregate effects of their downstream effectors, effectively collapsing lower-level regulatory complexity into higher-level control modules. Explicit inclusion of numerous downstream factors would introduce redundancy, increase model complexity, and reduce both computational tractability and interpretability. Importantly, despite this abstraction, the simulation results remain in good agreement with existing experimental data and recapitulate key biological phenotypic transitions. Therefore, upstream core regulators were prioritized as key components of the network.

The regulatory factors included in the model also exhibit cell state specificity. Myelinating, non-myelinating (Remak), and repair Schwann cells are characterized by distinct molecular expression profiles. Accordingly, within the differentiation module, factors with minimal overlap across these cell states were preferentially selected, thereby enabling clear discrimination of cellular phenotypes based on their simulated expression signatures.

Finally, the selection of regulatory factors is problem-oriented. As this study focuses on Schwann cell dedifferentiation, priority was given to key regulators driving cellular reprogramming. In the following section, we describe the construction of the network in detail.

### 2.3. Results of the Construction of the Endogenous Regulatory Network

The differentiation module is defined based on specific molecular markers associated with myelinating, non-myelinating, and reparative Schwann cells. In myelinating Schwann cells, the transcription factor Sox10 directly induces the key regulator Krox20, which is essential for peripheral myelin formation and thereby governs Schwann cell myelination [49,50]. Non-myelinating Schwann cells ensheath multiple small-caliber axons and express several molecular markers that are also present in immature Schwann cells, including neural cell adhesion molecule 1 (NCAM) and L1 cell adhesion molecule (L1CAM) [51,52]. After peripheral nerve injury, both myelinating and non-myelinating Schwann cells transdifferentiate into phenotypes that specifically promote nerve repair, providing metabolic support and guidance cues for neuronal survival, axonal regeneration, and target organ reinnervation. In the early stage, Schwann cells rapidly downregulate myelin-associated genes while activating repair-associated genes, such as c-Jun, Stat3, and Notch, to initiate dedifferentiation, and re-express cell adhesion molecules including NCAM and L1CAM. Subsequently, the expression of neurotrophic factors, such as BDNF, increases [3,53]. These molecules were collectively incorporated into the Schwann cell differentiation module.

**Figure 1:**
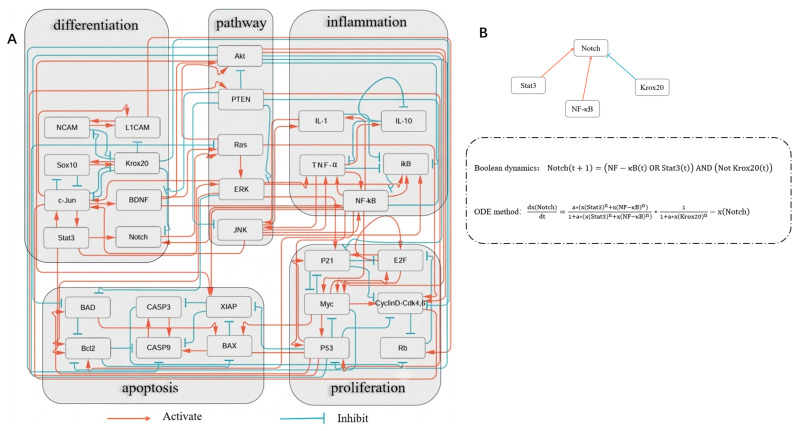
(**A**) The core endogenous molecular-cellular network underlying Schwann cell dedifferentiation comprises 30 nodes, 61 activating edges, and 43 inhibitory edges. Specifically, orange edges denote activation, while blue edges represent inhibition. Nodes are further clustered into distinct groups based on modular classification. (**B**) The endogenous network was modeled using both Boolean dynamics and differential equation–based approaches. As an illustrative example, the governing equation for the Notch node is presented. In this framework, x denotes the molecular concentration of the node, n is the Hill coefficient, and a represents the reciprocal of the apparent dissociation constant. The degradation rate is fixed at 1 in all models, corresponding to a linear decay term of −x. The complete gene interaction relationships are provided in Table S2 in Part A of the Supplementary Materials.

During the transition toward a reparative phenotype, Schwann cells also participate in an inflammatory response, upregulating cytokines such as tumor necrosis factor-α (TNF-α), nuclear factor kappa B (NF-κB) and interleukin-1β (IL-1β), while also producing anti-inflammatory mediators such as interleukin-10 (IL-10) and inhibitor of nuclear factor kappa B (iκB) to modulate the local immune environment and support nerve repair [3,29]. In the peripheral nerves of adult animals, Schwann cells remain in a quiescent state. Following injury, peripheral nerves undergo Wallerian degeneration, during which Schwann cells activate a proliferation program to facilitate nerve repair. At this stage, Schwann cell proliferation depends on cyclin D1. Notably, during embryonic development, D-type cyclins appear to be functionally redundant for Schwann cell growth. The canonical cell-cycle regulatory circuitry, comprising Cyclin D–cyclin-dependent kinase 4/6 complex (Cyclin D-Cdk4,6), RB transcriptional corepressor 1 (Rb), E2F transcription factor (E2F), MYC proto-oncogene, bHLH transcription factor (Myc), p53, and Cyclin dependent kinase inhibitor 1A (p21), plays a central role in coordinating cell-cycle progression and checkpoint control. In this regulatory framework, Cyclin D/Cdk4,6 promotes Rb phosphorylation, thereby releasing E2F-mediated transcriptional activity, whereas p53 contributes to cell-cycle checkpoint control primarily through the induction of p21 and subsequent inhibition of cyclin-dependent kinase activity. Therefore, we define the canonical cell cycle regulatory pathway, comprising Cyclin D-Cdk4,6, Rb, E2F, Myc, p53, and p21, as the proliferation module within the endogenous regulatory network of Schwann cells [32,33]. It should be noted that some components of this module, particularly p53 and E2F1, also participate in other biological processes such as apoptosis. Their inclusion in the proliferation-related module reflects their roles within the cell-cycle regulatory framework rather than exclusive functional specificity. However, because of the slow pace of axonal growth during regeneration, the repair phenotype of Schwann cells is inherently unstable. Over prolonged periods of regeneration, Schwann cells undergo regression of the repair phenotype and may enter programmed cell death. Therefore, in the constructed endogenous network of Schwann cells, we incorporated the factors BCL2 associated agonist of cell death (BAD), X-linked inhibitor of apoptosis (XIAP), Caspase 9 (CASP9), Caspase 3 (CASP3), BCL2 apoptosis regulator (Bcl2), and BCL2-associated X protein (BAX) to establish an apoptosis module [4,34,54]. Finally, following nerve injury, multiple signaling pathways in Schwann cells are activated in response to the insult, including the JNK, Ras/ERK, and PI3K/Akt pathways of the MAPK family [8,29,35]. The constructed endogenous regulatory network of Schwann cells comprises 30 nodes, 61 activating edges, and 43 inhibitory edges. In this network, nodes typically represent related genes, molecules, or signaling pathways. The network has no absolute upstream or downstream agents. To facilitate the representation of the network structure and enable subsequent dynamical modeling, the complex regulatory relationships were appropriately abstracted and simplified. Based on existing experimental evidence, the interactions between nodes were uniformly categorized into two types: activation and inhibition. Specifically, if one molecule promotes the production or activation of another, it is defined as an activation interaction. Conversely, if a molecule suppresses the expression of another or induces its inactivation, it is classified as an inhibitory interaction. In addition, the regulatory network is formulated as a coarse-grained dynamical system, in which each node represents an integrated functional state that captures the combined effects of transcriptional, translational, and post-translational regulation in a unified manner. Detailed regulatory relationships among all factors are provided in Table S2 in Part A of the Supplemental Material. The supporting references are listed in References [31,43,50,55,56,57,58,59,60,61,62,63,64,65,66,67,68,69,70,71,72,73,74,75,76,77,78,79,80,81,82,83,84,85,86,87,88,89,90,91,92,93,94,95,96,97,98,99,100,101,102,103,104,105,106,107,108,109,110,111,112,113,114,115,116,117,118,119,120,121,122,123,124,125,126,127,128,129,130,131,132,133,134,135]. The interactions among these factors are shown in Figure 1.

### 2.4. Mathematical Methods for Quantifying Endogenous Networks

#### 2.4.1. Boolean Dynamics

Boolean dynamics provides a computational framework that can capture the structural information of a network without focusing too much on its details [136,137,138]. In this framework, Notch nodes can be represented as:

$$Notch\left(t+1\right)=\left(NF-\kappa B\left(t\right)\;OR\;Stat3\left(t\right)\right)\;AND\;\left(Not\;Krox20\left(t\right)\right)$$

The equation forms of other factors are provided in Supplementary Section B. Boolean dynamics provides an effective method to capture the global structural characteristics of networks, as shown in Figure 1B. We adopted this model when constructing the endogenous network and compared it with the stochastic differential equation (SDE) method, which can identify saddle points that Boolean dynamics cannot recognize. It is worth noting that the modeling results of the core endogenous network are basically consistent between Boolean and differential equation representations, emphasizing that the dynamic behavior of the system is mainly determined by its intrinsic topology.

#### 2.4.2. Stochastic Differential Equation

Compared with simplified Boolean dynamics and computationally demanding master equations, stochastic differential equations (SDEs) preserve model details while maintaining controllable computational complexity [138,139]. In endogenous networks, the expression levels or activities of n factors are represented as **x** = (x_1_, x_2_, …, x_n_)^T^. We employ SDEs to describe the temporal evolution of these factors, and the general form of the stochastic differential equation can be expressed as follows:

$$\frac{dx_{i}}{dt}=f_{i}\left(x,\alpha \right)+\zeta_{i}\left(x,t\right)$$

Among them, $f_{i}\left(x,\alpha \right)$ is a nonlinear function parameterized by α, and ζ represents a multiplicative Gaussian white noise with a mean of zero. The correlation of the noise term is given by $\left\langle \zeta_{i}\left(x,t\right)\zeta_{j}\left(x,t^{\prime }\right)\right\rangle =2\epsilon D_{ij}\left(x\right)\delta \left(t-t^{\prime }\right)$. Here $\left\langle \ldots \right\rangle$ is the average distribution of noise, ϵ denotes the intensity of noise, $D_{ij}\left(x\right)$ is the diffusion coefficient matrix, and $\delta (t-t^{\prime })$ is the Dirac delta function. We have developed a framework for analyzing stochastic differential equations [140,141,142], in which the original equation can be decomposed into three components: a dissipative matrix $S\left(x\right)$, an antisymmetric matrix $A\left(x\right)$, and a potential function $\phi \left(x,\alpha \right)$,

$$\sum_{j}\left[S_{ij}\left(x\right)+A_{ij}(x)\right]\frac{dx_{j}}{dt}=-\frac{\partial \phi \left(x,\alpha \right)}{\partial x_{i}}+\xi_{i}\left(x,t\right)$$

where ξ is the zero-mean white noise with covariance satisfying $\left\langle \xi_{i}\left(x,t\right)\xi_{j}\left(x,t^{\prime }\right)\right\rangle =2\epsilon S_{ij}\left(x\right)\delta \left(t-t^{\prime }\right)$. By setting the stochastic and deterministic terms equal respectively, two relationships were obtained:

$$\sum_{s,t}\left[S_{is}\left(x\right)+A_{is}\left(x\right)\right]D_{st}\left(x\right)\left[S_{tj}\left(x\right)-A_{tj}\left(x\right)\right]=S_{ij}(x)$$

$$\sum_{j}\left[S_{ij}\left(x\right)+A_{ij}\left(x\right)\right]f_{j}\left(x\right)=-\partial_{i}\phi (x,\alpha )$$

The potential function can be derived using $\phi \left(x\right)=-\int \left[S\left(x\right)+A\left(x\right)\right]f\left(x\right)dx$. This connects the potential function ϕ(x,α) to the Boltzmann–Gibbs distribution $\rho \left(x\right)$ of the stochastic process in state space:

$$\rho \left(x\right)\propto exp\left[-\frac{\phi (x,\alpha )}{\epsilon }\right]$$

Because the system’s steady-state distribution follows the Boltzmann–Gibbs distribution, the potential function is decoupled from the noise intensity. Consequently, in our method, the potential function remains stable and invariant under any noise level. The existence of this potential function underpins our subsequent analysis.

In subsequent analyses, we adopt the method introduced above for evaluating endogenous networks, which effectively eliminates the dependence of Equation on noise. Focusing on the deterministic term of the equation $f_{i}\left(x,\alpha \right)$, we use the Hill function to describe how other factors exert activating or inhibitory influences on the expression level of the i-th agent [143]:

$$\frac{dx_{i}}{dt}=\frac{\sum_{u}a_{iu}\cdot x_{u}^{n_{iu}}}{1+\sum_{u}a_{iu}\cdot x_{u}^{n_{iu}}}\cdot \frac{1}{1+\sum_{v}a_{iv}\cdot x_{v}^{n_{v}^{iv}}}-\frac{x_{i}}{\tau_{i}}$$

Here, n denotes the Hill coefficient, a is the reciprocal of the apparent dissociation constant, $\sum_{u}a_{iu}\cdot x_{u}^{n_{iu}}$ and $\sum_{v}a_{iv}\cdot x_{v}^{n_{v}^{iv}}$ represent the cumulative activating and inhibitory influences on factor i, respectively, and $\tau_{i}$ denotes the degradation time of factor i, the equation for the Notch node, provided as an example, is shown in Figure 1B. Because precise estimation of these parameters across several orders of magnitude is challenging, we normalized the expression or activation levels of all factors to a range between 0 and 1. Previous studies have shown that biological systems possess strong intrinsic robustness, such that moderate variations in kinetic parameters generally do not alter their qualitative behaviors. Instead, the stability and adaptive characteristics of biological systems are largely governed by the topology of the underlying regulatory network [18,144]. To evaluate the robustness of the present framework, we examined multiple feasible mathematical formulations of the regulatory interactions and allowed model parameters, including a and n, to vary within biologically reasonable ranges. Under these different equation forms and parameter settings, the major steady states identified by the model remained consistently preserved. These results indicate that the principal dynamical characteristics of the system are governed predominantly by the underlying network topology rather than by specific parameter choices [25,27,145]. In subsequent calculations, all τ_i_ values were set to 1 for simplicity, assuming that factor degradation is primarily driven by cell division. In biochemical reactions, the Hill coefficient is typically in the range of 2–5 [146]. Accordingly, we chose a value of 3 for all activation and inhibition interactions in our model. Positioning the steep ‘S’ region of the sigmoid function near the center ensures that the network is most sensitive to input variations around the baseline, facilitates controllable steady states, and better captures threshold-like decision behaviors characteristic of biological systems. Accordingly, the parameters *a* and *n* satisfy the relationship $a\approx 2^{n}$. Here, we set a = 10 and n = 3. Each factor in the network can be described as such differential equations, and all equations are provided in Part C of the Supplementary Materials.

### 2.5. Solution to ODE

We employ the Newton and Euler methods to solve nonlinear differential equations, using uniformly distributed random vectors $X_{0}$ as initial conditions:

The ODE system was simulated using an explicit Euler scheme with a fixed step size, and iterations continued until both the change between successive states and the magnitude of the vector field fell below predefined convergence thresholds, indicating convergence to a steady state. In parallel, fixed points of the Hill equations were directly identified using the MATLAB R2023a (MathWorks, Natick, MA, USA) function fsolve, which applies the Newton method. Solutions of $f\left(X\right)=0$ were classified by evaluating the Jacobian matrix: points with all eigenvalues having negative real parts were designated as stable fixed points, whereas those with one or more positive eigenvalues were identified as saddle points, with the number of unstable directions recorded; solutions with multiple positive eigenvalues were further defined as hypertransition states. To ensure robustness and avoid numerical bias, we applied both the Euler method and the Newton method concurrently, and only steady states consistently detected by both approaches were retained.

### 2.6. Perturbations on Endogenous Network

In general, disturbances to dynamical systems can be categorized into two major types: structural and positional perturbations. Structural perturbations manifest as modifications to the parameters or functional form of the governing equations, whereas positional perturbations arise from fluctuations in the system’s internal noise or changes in the external environment, such as drug-induced stimuli. Accumulated intrinsic noise can drive spontaneous transitions from one steady state to another via intermediate transition states, while external environmental shifts may directly displace the system from one basin of attraction to another.

In this study, we kept the governing equations unchanged and introduced small stochastic fluctuations to mimic noise-driven crossings of local energy barriers, enabling reconstruction of the transition pathways linking intermediate states to their adjacent steady states. This procedure allowed us to identify both the steady states and the corresponding transition trajectories shaped by the endogenous network structure. In addition, we implemented positional perturbations that emulate external interventions by modifying the initial expression levels of selected factors to mimic gene upregulation or downregulation, thereby simulating drug-induced environmental changes.

## 3. Results

### 3.1. Endogenous Network Modeling Results of Schwann Cells

Schwann cells undergo dedifferentiation to acquire a repair phenotype following peripheral nerve injury. To elucidate the underlying mechanisms, we constructed the core endogenous regulatory network of Schwann cells. The interactions among related modules and key signaling pathways are illustrated in Figure 1. As described previously, this network is closed, with each node both regulating and being regulated by others. There is no absolute upstream or downstream hierarchy within the system. The network operates autonomously and eventually converges to biologically meaningful steady states. The computational framework is detailed in the Materials and Methods section.

**Figure 2:**
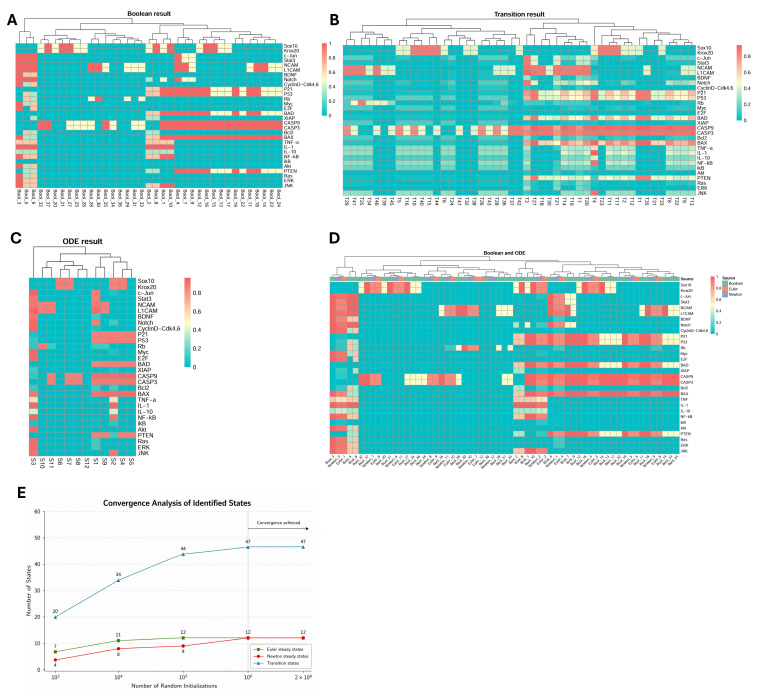
Endogenous networks of Schwann cells were quantified via differential equations and Boolean algebra. (**A**) A total of 37 attractors were computed using Boolean algebra with one million random initial values. (**B**) Forty-seven transition states were derived via Newton’s method following one million random vector iterations. (**C**) Twelve steady states were solved using ordinary differential equations (ODEs). (**D**) Comparative analysis of attractors from Boolean algebra versus steady states and transition states obtained via ODEs. The specific numerical values for each steady state are provided in the Part D of the Supplementary Materials. (**E**) Number of steady states calculated by different differential equation methods under varying random initial vectors. The plot shows the number of steady states obtained via Newton’s method and Euler’s method across different numbers of random initial vectors. The *x*-axis represents the number of random initial vectors, and the *y*-axis represents the number of obtained steady states or transition states. Symbols indicate different states and methods: blue triangles denote transition states, green squares denote results from Euler’s method, and red circles denote results from Newton’s method.

The system was modeled using a set of high-dimensional nonlinear differential equations. To evaluate whether the number of random initial vectors was sufficient for identifying the attractor landscape, a convergence analysis was performed by progressively increasing the number of random initial vectors from 10^3^ to 2 × 10^6^. As shown in Figure 2E, the number of identified steady states increased with increasing sampling size at lower initialization numbers but gradually approached saturation. Specifically, the Euler method identified 7, 11, and 12 steady states at 10^3^, 10^4^, and 10^5^ initializations, respectively, and remained unchanged thereafter. Similarly, the Newton method identified 4, 8, 9, and 12 steady states at 10^3^, 10^4^, 10^5^, and 10^6^ initializations, respectively. Importantly, no additional steady states were detected when the number of random initial vectors was further increased from 10^6^ to 2 × 10^6^. Likewise, the number of transition states increased from 20 to 47 and remained unchanged between 10^6^ and 2 × 10^6^ initializations, as shown in Figure 2E. These results indicate that both the steady-state and transition-state landscapes had effectively converged, supporting the sufficiency of the selected sampling size and the completeness of the identified network states. As shown in Figure 2B, the network contains 47 identified transition states, whereas Figure 2C illustrates the 12 steady states obtained from the ODE-based analysis. In the Boolean dynamic simulations, we tested one million distinct initial conditions and identified 37 attractors, comprising 7 point attractors and 30 linear attractors, as illustrated in Figure 2A. Comparison with the ODE results showed that each Boolean attractor had a corresponding ODE solution, with the linear attractors representing transition states, as shown in Figure 2D. As described in the Materials and Methods section, the agreement between the two modeling approaches further supports the reliability of the identified attractor landscape. Detailed computational results for Boolean dynamics and differential equations are presented in Tables S3–S5 in Part D of the Supplementary Materials. The complete results of the convergence analysis, including all state-identification outcomes obtained from different numbers of random initial vectors, are provided in a separate Supplementary Excel file.

### 3.2. Steady States Correspond to Different Cell Types

To further investigate the potential biological significance of the steady states generated by the endogenous regulatory network, we first analyzed the biological implications of each steady state at the module level. Specifically, the status of each module was determined by examining the expression levels of factors within the corresponding functional modules in each steady state. In this study, a threshold of 0.5 was first set, with factor expression levels above 0.5 considered active (corresponding to 1) and those below 0.5 considered inactive (corresponding to 0). Accordingly, the expression levels of all factors were categorized into active and inactive states. Taking the cell cycle module as an example, we further illustrate how the functional state of a module under a steady state is determined based on the expression levels of its key factors. For each steady state, the status of each module was defined as either ON or OFF.

After thresholding, the expression levels of factors in the cell cycle module under steady state S3 are as follows:

Cyclin D-Cdk4,6 = 1, P21 = 0, P53 = 0, Rb = 0, Myc = 1, E2F = 1,

In steady state S3, the expression pattern of the cell cycle module exhibits a pro-proliferative signature, indicating its active state, as the activation of Cyclin D–Cdk4/6 promotes G1-to-S phase progression, Myc and E2F enhance cell cycle gene expression, and low levels of P21, P53, and Rb reduce inhibitory regulation. Similarly, the expression states of other modules were defined using the same approach. In particular, lineage-specific factors within the differentiation module were used to preliminarily define cell types, and the detailed expression values of all factors in each steady state are provided in Supplementary Material C. Based on the expression patterns of factors in the differentiation module, the 12 steady states were first classified into four categories, representing different Schwann cell types and functional states, as summarized in Table 1. Based on existing experimental evidence, the expression characteristics of myelinating, non-myelinating, and repair Schwann cell states across different functional modules are summarized in Table 2. Accordingly, we further determined which specific cell type each steady state represents.

In steady state S6, the myelin-associated transcription factors Krox20 and Sox10 are highly expressed. Within the proliferation module, CyclinD–Cdk4/6 expression is suppressed, indicating that cells are in a quiescent cell cycle state. In the apoptosis module, Casp3 and Casp9 are not expressed, suggesting that the apoptotic program is not activated. In addition, inflammatory factors are absent, indicating a lack of inflammatory response, and major signaling pathways remain inactive. In a study comparing the functions of mutant and wild-type Krox20, the latter was shown to promote Schwann cell exit from the cell cycle and suppress apoptosis [147]. This is consistent with the characteristics of mature myelinating Schwann cells in intact nerves, and therefore we define S6 as representing the myelinating Schwann cell. Using the same analytical framework, we next examined other steady states. In steady state S10, NCAM and L1CAM are highly expressed, whereas myelin-associated and dedifferentiation-related factors are suppressed. The cell cycle remains in a quiescent state, with no expression of Casp3 and Casp9, indicating that apoptosis is not occurring. Inflammatory factors and signaling pathways are also suppressed. These features are consistent with the known characteristics of non-myelinating Schwann cells, particularly their association with NCAM and L1CAM expression [4,148]. Therefore, S10 is defined as the non-myelinating Schwann cell. In steady state S3, myelin-associated factors are suppressed, while dedifferentiation-related factors, including c-Jun, Stat3, and BDNF, are markedly upregulated. The increased expression of CyclinD–Cdk4/6 suggests that cells have re-entered the cell cycle and initiated proliferation. Meanwhile, Casp3 and Casp9 remain unexpressed, indicating that apoptosis is not activated. In addition, inflammatory factors such as TNF-α and IL-1 are activated, suggesting that the cells are undergoing an inflammatory response. Key signaling pathways, including JNK, Akt, and ERK, are also activated. These molecular features are consistent with experimentally reported characteristics of reparative Schwann cells, which exhibit downregulation of myelin-associated genes, upregulation of dedifferentiation-related factors, activation of inflammatory responses, and increased proliferative activity. Importantly, as no apoptotic program is engaged in this state, S3 corresponds to a normal reparative Schwann cell phenotype [3,149,150]. In the following sections, steady state S6 will be referred to as the “M-steady state” (myelinating, M), S10 as the “NM-steady state” (non-myelinating, NM), and S3 as the “RSC-steady state” (repair Schwann cells, RSC).

Based on the previous analytical approach, we further examined the biological significance of other steady states. As shown in Figure 2C, steady state S1 is characterized by relatively high expression of key repair-associated regulators, including c-Jun, Stat3, and Notch, indicating partial preservation of the repair program. However, the neurotrophic factor BDNF is expressed at very low levels, accompanied by suppressed cell-cycle activity and reduced signaling pathway activity, indicating a general decline in the maintenance of the reparative phenotype. Notably, apoptotic signaling is activated in this state, suggesting the emergence of a pro-apoptotic tendency despite the partial retention of repair-associated transcriptional programs. Together, these features are indicative of a Schwann cell state with reduced reparative capacity following prolonged denervation, reflecting an incomplete or insufficiently sustained repair phenotype rather than full functional regeneration. This interpretation is consistent with experimental observations showing that neurotrophic factors such as BDNF and GDNF exhibit a transient increase followed by a progressive decline during prolonged denervation in the absence of sustained regenerative support, which correlates with diminished neuronal survival and limited axonal regeneration, reflecting a gradual deterioration of the repair-supportive microenvironment [151,152,153]. In contrast, steady state S2 is characterized by markedly reduced expression of both neurotrophic and dedifferentiation-associated factors, together with elevated inflammatory signaling and enhanced expression of pro-apoptotic regulators, including Casp3 and Casp9, as well as widespread suppression of signaling pathways. Compared with S1, this state exhibits a more pronounced loss of repair-associated features and a stronger apoptotic signature. Such a profile is consistent with an inflammation-associated, apoptosis-prone dysfunctional Schwann cell state that may arise under chronic denervation conditions, in which persistent inflammatory stress further compromises regenerative capacity. Previous studies have shown that chronic inflammation can disrupt Schwann cell repair functions and impair their ability to support nerve regeneration. Therefore, S2 may represent a more advanced stage of repair failure characterized by combined inflammatory dysregulation and apoptosis-prone signaling [152,154]. Because both S1 and S2 exhibit reduced reparative capacity compared with the canonical repair Schwann cell state, they are collectively referred to as the “DRSC-steady state” (declining repair Schwann cell, DRSC), reflecting their diminished reparative capacity. However, this grouping is intended as a functional classification rather than implying biological equivalence. The molecular profiles suggest that S1 and S2 reflect distinct manifestations of repair decline. S1 is more consistent with a reduced-reparative-capacity state in which key repair-associated regulators such as c-Jun, Stat3, and Notch remain partially active, but neurotrophic support (e.g., BDNF) and downstream signaling activity are insufficient to sustain a fully functional regenerative phenotype. In contrast, S2 represents an inflammation-associated dysfunctional Schwann cell state characterized by elevated inflammatory signaling and increased expression of pro-apoptotic factors, including Casp3 and Casp9, together with a more pronounced loss of repair-associated features.

Finally, in steady states S4, S5, S7, S8, S9, and S11, the apoptosis module is activated, accompanied by high expression levels of Casp3 and Casp9, while other modules and signaling pathways remain at low expression levels, indicating that the cells are predominantly executing the apoptotic program. In steady state S12, all factors are expressed at negligible levels. Therefore, these steady states were classified as apoptotic cells, and hereafter are collectively referred to as the “ASC-steady state” (apoptotic Schwann cell, ASC). This classification is based on shared apoptotic features, despite minor differences in molecular expression patterns among these steady states.

### 3.3. Model Validation Using Published Datasets

To further validate the biological relevance of the steady states derived from the mathematical model, we integrated multi-dimensional experimental evidence by systematically comparing the predicted expression patterns of key factors with both low-throughput experimental data and high-throughput transcriptomic datasets. Notably, validation primarily focused on reparative Schwann cells, based on two considerations. Firstly, reparative Schwann cells exhibit well-defined molecular characteristics in nerve injury models, with relatively abundant data available, ensuring the reliability of the validation benchmark. Second, mature myelinating and non-myelinating Schwann cells often exhibit overlapping transcriptional features and remain challenging to distinguish or purify in existing transcriptomic datasets, as reported in previous studies [15]. Therefore, the experimental validation was mainly conducted on reparative Schwann cells, evaluating the model predictions at the level of key molecular expression, thereby achieving a balance between data availability and analytical reliability. Importantly, no experimental datasets were used as direct inputs during network construction or dynamical calculations. The endogenous regulatory network was established entirely based on experimentally reported regulatory relationships, and all steady states were obtained through nonlinear dynamical computations. Therefore, the resulting steady states represent purely theoretical predictions rather than data-constrained outcomes. Consequently, all comparisons with experimental datasets constitute independent validations of the model predictions.

**Figure 3:**
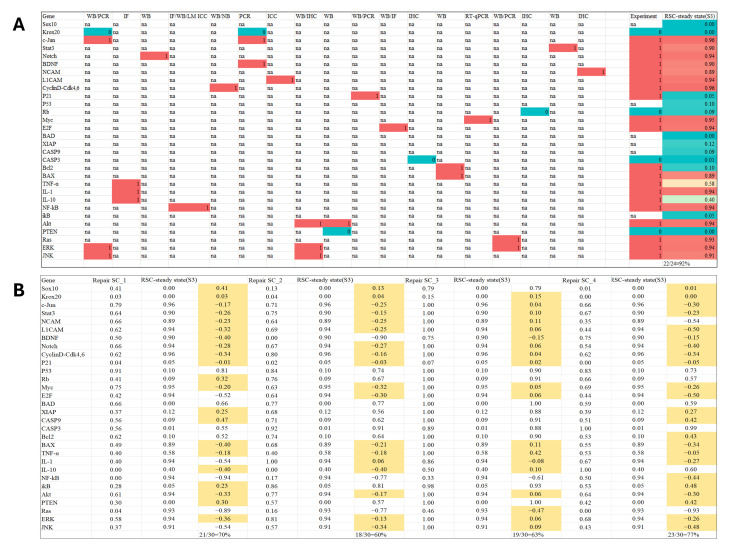
Experimental validation of the endogenous network computational results. (**A**) Based on existing knowledge from low-throughput experimental data, the expression profiles of multiple genes in Schwann cells following differentiation were systematically collated. Each column of the table denotes one independent study or experimental dataset. The “na” indicates that the corresponding factor was not included or investigated in the respective low-throughput study. Red color indicates high expression, whereas turquoise indicates low expression. (**B**) Transcriptomic data of peripheral nerve injury models were retrieved from the public Gene Expression Omnibus (GEO) database (accession number: GSE109075). After preprocessing data from injured samples and control group samples, the transcriptomic data were normalized to the range of 0–1. In (**B**), highlighted cells indicate that the absolute difference between the normalized transcriptomic expression level and the simulated value of the corresponding factor is ≤0.5. Normalization methods for high-throughput data are provided in the Part E of Supplementary Materials.

As an initial step of experimental validation, we compared the predicted expression patterns of the RSC-steady state (S3) with evidence derived from low-throughput molecular experiments. Techniques such as quantitative polymerase chain reaction (PCR), Western blotting, and immunohistochemistry (IHC) are widely accepted approaches for validating gene and protein expression changes and provide direct experimental evidence at the molecular level. Therefore, agreement between model predictions and observations obtained from these methods can serve as a reliable indicator of the biological relevance of the predicted steady state. Experimental evidence was collected from studies employing these commonly used low-throughput approaches [11,12,32,105,128,155,156,157,158,159,160,161,162,163,164,165,166,167]. Because these methods primarily provide qualitative or semi-quantitative information, the purpose of this validation was to evaluate whether the predicted changes in factor expression between normal Schwann cells and repair Schwann cells were consistent with experimentally reported trends following peripheral nerve injury, rather than to compare absolute quantitative expression levels. For experimental data, if a target factor is reported as significantly upregulated, strongly positive, or highly expressed in the literature or experimental observations, it is assigned as an active state with a value of 1. Conversely, if it is observed to be lowly expressed or undetectable, it is assigned as an inactive state with a value of 0. Factors lacking publicly available supporting data, as well as those not reported in a given study, were assigned a value of “na”. For the steady states derived from the model, factor expression levels were normalized to the range of 0–1. A threshold of 0.4 was adopted to discretize the steady-state values into low- and high-activity states. Values greater than 0.4 were assigned to 1, whereas values below 0.4 were assigned to 0. This threshold was selected as a balanced criterion within the normalized range: substantially lower thresholds would tend to classify a large proportion of factors as highly expressed, whereas substantially higher thresholds would classify only strongly activated factors as active. Therefore, 0.4 was chosen to distinguish relatively high and low activity states while avoiding overly permissive or overly stringent classification. Except for several factors for which experimental data were unavailable, the RSC steady state (S3) showed agreement with 22 of the 24 comparable factors (92%) reported in low-throughput experimental studies, supporting the reliability of the model predictions (Figure 3A). To assess the sensitivity of this result to threshold selection, we additionally applied a more stringent threshold of 0.5. Under this criterion, 21 of the 24 comparable factors remained consistent with experimental observations (approximately 88%), with only a single factor (IL-10) changing its classification status relative to the original threshold, reflecting that its predicted activity level was close to the threshold boundary. This finding indicates that the overall validation outcome is robust to reasonable variations in threshold selection. The references for the low-throughput data can be found in Part E of the Supplementary Material.

Although low-throughput experiments provide direct molecular evidence and allow accurate characterization of specific factors, they typically focus on a limited number of genes or proteins and therefore cannot comprehensively evaluate all components of the regulatory network. We assume a positive correlation between mRNA expression and protein levels for the core factors in the endogenous regulatory network. To achieve a broader and more systematic assessment of the model predictions, we further validated the calculated steady states using high-throughput transcriptomic datasets. The dataset GSE109075 was selected, which profiles the transcriptomes of normal adult sciatic nerves and injured nerves. The data were normalized to a range of 0–1, where 0 represents the lowest expression level of a factor and 1 represents the highest. A threshold of 0.5 was adopted as a conservative agreement criterion. Specifically, if the absolute difference between the model-predicted value and the normalized experimental value satisfied |Model − Data| ≤ 0.5, the factor was considered consistent; otherwise, it was considered inconsistent. This criterion corresponds to half of the normalized expression range and provides a balanced trade-off between overly stringent and overly permissive matching criteria when comparing idealized theoretical attractor states with biologically heterogeneous transcriptomic measurements. As illustrated in Figure 3B, the overall consistency between the calculated steady states and the high-throughput data ranged from 60% to 77%. Considering that transcriptomic data inherently contain biological variability and technical noise, and that the model predictions correspond to idealized steady states rather than individual biological samples, this level of agreement is reasonable and within expectations [168,169]. Moreover, since no transcriptomic datasets were used during network construction or dynamical calculations, this result provides independent support for the reliability and predictive capability of the model.

To further evaluate the biological relevance of the predicted attractor landscape, we compared the computed steady states (M-steady state, NM-steady state, and RSC-steady state) with single-cell RNA sequencing (scRNA-seq) data. We utilized a rat sciatic nerve injury dataset from GSE216665, which spans uninjured (naive) nerves as well as 3, 12, and 60 days post-injury. Data analysis was performed using the Seurat (v4) R package, with detailed procedures provided in Part F of the Supplementary Methods. Unlike low-throughput and bulk transcriptomic validations that primarily assess individual factors, this scRNA-seq analysis evaluates higher-level system behavior. Specifically, it tests whether the observed cell distributions match the predicted attractors, and whether their differentiation trajectories follow the theoretical transitions.

**Figure 4:**
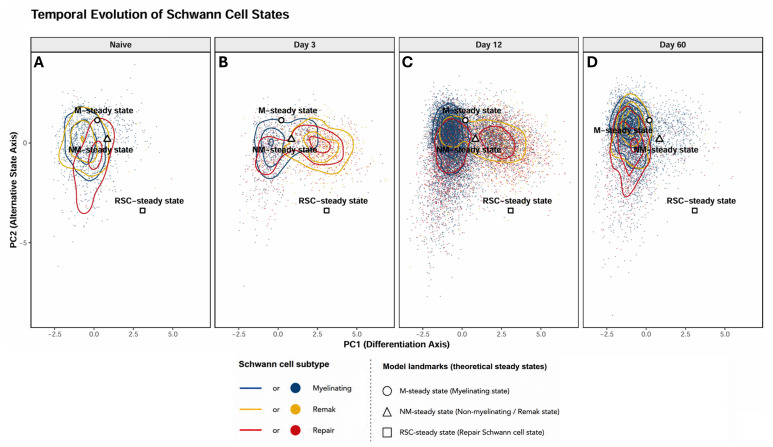
Temporal trajectory of Schwann cell phenotypic evolution projected onto the 30-node regulatory state space. (**A**–**D**) Principal Component Analysis (PCA) projection of single-cell transcriptomic profiles (GSE216665) from rat sciatic nerves at four stages: Naive, Day 3, Day 12, and Day 60 post-injury. Each dot represents an individual cell, color-coded by the inferred Schwann cell subtype: myelinating (blue), Remak/non-myelinating (yellow), and repair Schwann cells (red). Density contours indicate the spatial distribution and concentration of each subtype across the regenerative timeline. Theoretical steady-state landmarks derived independently from the mathematical regulatory model are superimposed onto the transcriptomic state space. These landmarks include the myelinating steady state (M-steady state, open circle), non-myelinating steady state (NM-steady state, open triangle), and repair Schwann cell steady state (RSC-steady state, open square). Detailed analytical procedures are provided in Part F of the Supplementary Information. The projection reveals dynamic temporal transitions of Schwann cell populations during nerve regeneration, demonstrating strong concordance between experimentally observed transcriptional states and theoretically predicted attractor states.

As shown in Figure 4, the colored contour lines represent the density distribution of real single cells, whereas the black open symbols denote independently computed theoretical steady states. In the uninjured condition, the dark blue (myelinating) and yellow (Remak) cell populations are tightly clustered on the left side of the PC1 axis and closely correspond to the theoretically predicted M-steady state and NM-steady state, indicating that the model accurately captures the characteristic features of healthy Schwann cell states. Following injury, a large population of red cells representing repair Schwann cells emerges and extends toward the lower-right region of the state space, where the model-predicted RSC-steady state is located. Notably, in the day 12 dataset, the distribution of repair Schwann cells shows a clear directional tendency toward the predicted RSC attractor, suggesting consistency between the experimentally observed activation of the repair program and the repair-associated state identified by the model. Within our computational framework, the RSC-steady state represents an idealized attractor corresponding to the theoretical limit of the repair phenotype rather than a specific post-injury time point. In the day 60 dataset, the repair Schwann cell population is markedly reduced and exhibits a tendency to retract from the repair-associated region. Such changes are compatible with biological processes known to occur during prolonged denervation, including a decline in the maintenance of the repair phenotype, as well as the potential redifferentiation of repair Schwann cells toward mature Schwann cell states following successful regeneration. Importantly, the upper-left region of the state space corresponds to the mature Schwann cell attractors predicted by the model. Therefore, the observed reduction in repair Schwann cells together with their redistribution toward this region is broadly consistent with the state-transition tendencies inferred from the attractor landscape. Taken together, these results do not imply a direct correspondence between model states and specific biological time points. They indicate that the experimentally observed progression of Schwann cell populations across different stages of denervation is broadly consistent with the state-transition tendencies predicted by the model. Therefore, the scRNA-seq analysis provides support for the biological relevance of the predicted attractor landscape and its associated transition relationships.

In summary, the convergence of evidence from low-throughput experiments, bulk transcriptomic datasets, and single-cell RNA sequencing analyses supports the biological relevance and reliability of the proposed endogenous regulatory network. These three validation strategies assess the model from complementary perspectives, including factor-level expression patterns, transcriptome-wide profiles, and cell-state transition dynamics. The overall agreement observed across these independent datasets provides strong support for the predictive capability of the proposed dynamical framework.

### 3.4. Landscape of Schwann Cell Dedifferentiation

**Figure 5:**
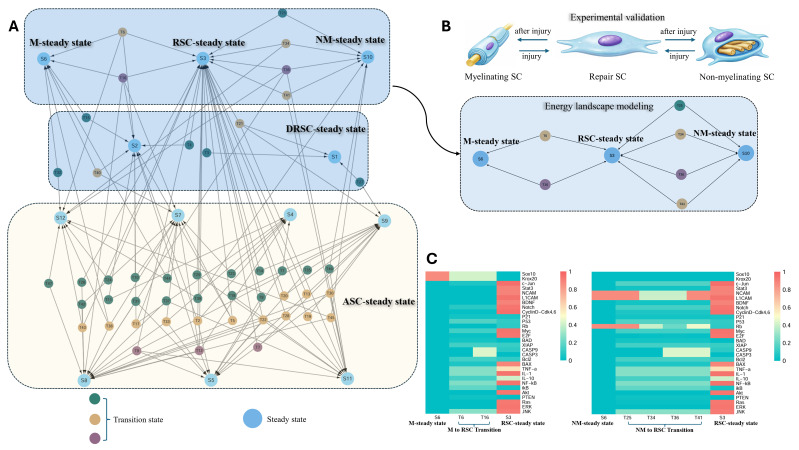
(**A**) In the first blue box, steady state S6 corresponds to myelinating Schwann cells, S3 represents repair Schwann cells, and S10 denotes non-myelinating Schwann cells. These phenotypic states are interconnected through multiple transition states (T6, T16, T25, T34, T36, and T41), forming a structured dynamical landscape. The landscape derived from the endogenous network captures the dedifferentiation dynamics of Schwann cells following peripheral nerve injury. In the second blue box, steady states S2 and S1 represent Schwann cell states with reduced repair capacity. The steady state highlighted in the yellow box corresponds to Schwann cells exhibiting a propensity toward apoptosis. Arrows indicate transitions from transition states toward steady states. In panel A, smaller green, brown, and purple circles represent transition states, whereas larger blue circles represent steady states. (**B**) The upper panel summarizes experimentally validated dedifferentiation pathways of Schwann cells. The lower panel shows the cell state transition landscape obtained from endogenous network modeling (delineated by the blue box). The model-derived transitions are consistent with established experimental observations. (**C**) Mechanisms underlying changes in factor expression profiles during the transition of myelinating and non- myelinating Schwann cells to reparative Schwann cells.

To characterize the dedifferentiation behavior of Schwann cells following peripheral nerve injury, we further introduced perturbations into the network to derive the transition paths between stable and transition states. By connecting all stable and transition states, we reconstructed the overall network topology, as shown in Figure 5A.

Transition states were classified according to the number of eigenvalues of the Jacobian matrix with non-negative real parts, these transition states interconnect distinct steady states. After overcoming the potential barriers between them, different steady states can achieve mutual conversion. The resulting landscape map reveals the dedifferentiation pathways of Schwann cells, as highlighted by the first blue box in Figure 5A. Within this framework, the M-steady state (S6), NM-steady state (S10), and RSC-steady state (S3) are interconnected by multiple transition states. In the computed landscape, both the M-steady state (S6) and NM-steady state (S10) can transition to the RSC-steady state (S3), Figure 5B. This finding is particularly noteworthy, as reported by Jessen, both myelinating and non-myelinating Schwann cells can dedifferentiate into repair Schwann cells following peripheral nerve injury. Our computational results successfully recapitulate this phenotypic transition [3,49,170].

We further analyzed the transition states connecting the M-steady state (S6), NM-steady state (S10), RSC-steady state (S3). In the following sections, the transitional states connecting the M-steady state (S6) and the RSC-steady state (S3) are referred to as “M to RSC Transition”, while those connecting the NM-steady state (S10) and the RSC-steady state (S3) are referred to as “NM to RSC Transition”. Among them, the M to RSC Transitions (T6, T16) directly link the M-steady state (S6) to the RSC-steady state (S3). As shown in Figure 5C, we visualized the expression profiles of the M to RSC Transitions (T6, T16). Compared with the M-steady state (S6), both transitional states exhibit a prominent downregulation of myelin-associated factors, accompanied by an overall upregulation of inflammatory modules. In addition, the JNK signaling pathway becomes activated, while dedifferentiation-associated regulators, including c-Jun and Notch, show moderate increases. Proliferation-related factors are slightly upregulated but do not exhibit substantial changes overall, whereas pro-survival factors such as Bcl2 and XIAP display mild increases.

These molecular features are consistent with the well-established Schwann cell injury response program described by Jessen and colleagues. Following nerve injury, myelin-associated genes are rapidly downregulated, accompanied by the activation of proliferative activity and the re-expression of factors that are normally inactive in intact nerves, such as TNF-α, IL-1, and BDNF. Therefore, our results recapitulate key aspects of experimentally observed Schwann cell dedifferentiation [3].

Previous studies have shown that following delayed nerve repair, the number of Schwann cells that can be isolated from the distal nerve stump gradually decreases [163,171]. The observation of staged apoptotic events suggests that distinct Schwann cell subpopulations, characterized by different levels of stability and sensitivity to apoptosis, may undergo selective cell death at different time points. However, current studies on Schwann cell dedifferentiation have primarily focused on phenotypic reprogramming and regeneration-associated functions, with comparatively less emphasis on the apoptotic dynamics during intermediate transitional states. Notably, the apoptotic factors Casp3 and Casp9 exhibit higher activity states in the M to RSC Transition (T16) compared with M to RSC Transition (T6), while the activity levels of anti-apoptotic components remain relatively unchanged, suggesting a shift toward a higher apoptotic tendency in T16 at the module level. Based on this observation, we propose that repair Schwann cells derived from dedifferentiation may exhibit intrinsic heterogeneity. Specifically, transitional states such as M to RSC Transition (T16) may represent a subpopulation of Schwann cells that are more prone to apoptosis and therefore have a shorter lifespan compared with typical repair Schwann cells. The identification and targeted modulation of such vulnerable subpopulations may provide new opportunities for therapeutic intervention in peripheral nerve injury.

In non-myelinating Schwann cells, NM to RSC Transitions (T25, T34, T36 and T41) connect to the RSC-steady state (S3). We further analyzed these transitional states based on their expression profiles in Figure 5C. It can be observed that the four transitional states share several common features. Specifically, they exhibit a moderate upregulation of c-Jun and Notch, a marked increase in inflammation-related factors, and a gradual elevation in the expression of cell cycle–associated genes. In addition, pro-survival factors such as Bcl2 and XIAP are upregulated, accompanied by progressive activation of the JNK signaling pathway. Notably, NCAM and L1CAM remain highly expressed across all four transitional states. In myelinating Schwann cells, the upregulation of NCAM and L1CAM during dedifferentiation is likely attributable to the fact that these adhesion molecules are suppressed under normal myelinating conditions and are reactivated upon injury. In contrast, non-myelinating Schwann cells intrinsically maintain the expression of these molecules, making their sustained high levels in transitional states biologically plausible. This observation is consistent with the characteristic molecular profile of non-myelinating Schwann cells, which express adhesion molecules such as NCAM and L1CAM while lacking myelin-associated proteins. Previous immunohistochemical analyses of human peripheral nerve sections have shown that non-myelinating Schwann cells express NCAM but do not express myelin-forming proteins, further supporting the biological plausibility of our results [3,29,172]. Similar to the M to RSC transition (T16), elevated expression levels of casp9 and casp3 are also observed in the NM to RSC transitions (T36 and T41). This suggests that during the transition from non-myelinating Schwann cells to repair Schwann cells, intermediate states may also exhibit a certain degree of heterogeneity, with some transitional states potentially displaying a higher propensity for apoptosis.

**Figure 6:**
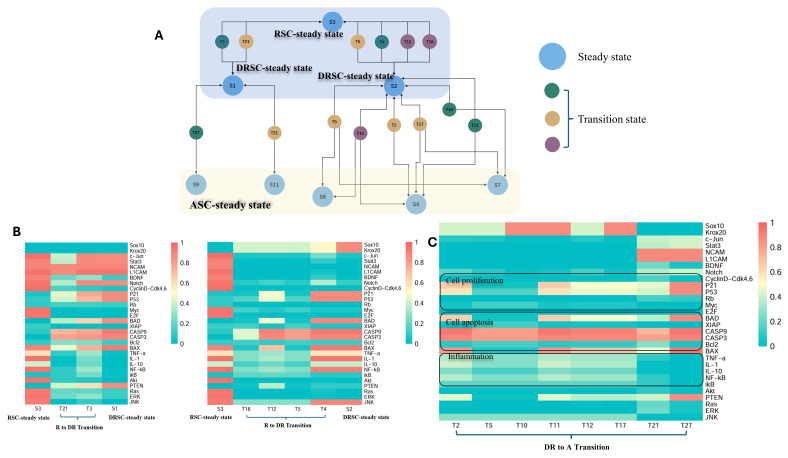
(**A**) The endogenous network predicts the trajectory by which repair Schwann cells (RSC) undergo a decline in reparative phenotype and gradually exhibit an apoptotic tendency. The blue box encompasses repair Schwann cells and those with a dysfunctional phenotype, whereas the yellow box denotes Schwann cells with an apoptotic propensity. Arrows indicate the potential differentiation directions of transition states. Smaller green, brown, and purple circles represent transition states, whereas larger blue circles represent steady states. (**B**) The transition process from repair Schwann cells to a state of dysfunctional phenotype, along with the underlying mechanisms governing changes in factor expression. (**C**) Factor expression profiles of transition states between dysfunctional Schwann cells and those with an apoptotic propensity.

In the previous section, we identified the steady states representing impaired repair capacity, namely the DRSC steady states (S1 and S2), and systematically characterized their expression profiles. Building upon this, we further investigated their relationship with the repair Schwann cell steady state (RSC steady state, S3). Within the endogenous regulatory network landscape constructed in this study, the transition states T4, T5, T12, and T16 connect the RSC steady state (S3) to the DRSC steady state (S2), describing a trajectory of progressive loss of repair capacity, as shown in Figure 6A. For clarity, these transition states are hereafter collectively referred to as the R to DR Transition. The expression levels of all regulatory factors across these transition states are visualized in Figure 6B. From the expression patterns, dedifferentiation-associated factors are consistently downregulated during the R to DR Transition (T4, T5, T12, and T16), while myelination-related factors are progressively upregulated. Notably, apoptosis-related factors, including casp3 and casp9, show a gradual increase in expression. In parallel, the cell cycle is suppressed, key signaling pathways become progressively inhibited, and inflammatory factors such as TNF-α and IL-1 remain persistently expressed. Biologically, these results indicate that the cells are not following a remyelination trajectory, but instead exhibit sustained inflammatory activation. Ultimately, in the DRSC steady state (S2), casp3 and casp9 reach markedly elevated levels, indicating activation of the apoptotic program. This prediction is consistent with previous experimental observations and reviews indicating that chronic inflammation can lead to a decline in repair capacity and apoptosis of Schwann cells [154]. For example, a spatio-temporal analysis of motoneuron survival, axonal regeneration, and neurotrophic factor expression following ventral root avulsion and reimplantation has shown that, under conditions of chronic denervation, the expression of neurotrophic factors fails to be sustained over time [153]. This finding supports our model prediction that Schwann cells with impaired repair capacity are unable to maintain a pro-regenerative molecular program. Importantly, recent reviews have indicated that chronic inflammation can lead to downregulation of BDNF. In our computational results, the transition from the RSC-steady state (S3) to the DRSC-steady state (S2) is accompanied by sustained inflammatory activation and suppressed BDNF expression, indicating that our network successfully captures this process [154].

In addition, as shown in Figure 6A, we analyzed the transition states T3 and T21 connecting the RSC-steady state (S3) and DRSC-steady state (S1), which we hereafter refer to as R to DR Transition (T3, T21). As shown in Figure 6B, these two transition states exhibit downregulation of BDNF, inhibition of the cell cycle, gradual upregulation of the apoptotic factors casp3 and casp9, very low expression of the anti-apoptotic factors Bcl2 and XIAP, and suppression of inflammation-related factors and signaling pathways, without signs of chronic inflammation. Notably, c-Jun is further downregulated in R to DR Transition (T21). Both transition states correspond to features of Schwann cells with declining repair capacity, resembling the long-term denervated Schwann cells. In a recent study using a mouse sciatic nerve transection model, SCs from aged animals and chronically denervated nerves were analyzed by immunofluorescence for markers of senescence. The analyses demonstrated that chronically denervated Schwann cells exhibit canonical features of repair capacity decline, such as irreversible cell cycle arrest. In a rat sciatic nerve transection model, the researchers observed a marked reduction in Schwann cell numbers in the distal stump after a 6-month delayed repair, indicating that prolonged denervation leads to a decline in Schwann cell repair capacity, potentially progressing to apoptosis [152,171,173,174].

Moreover, the landscape generated from quantifying the endogenous network predicts the transition by which repairing Schwann cells progressively lose their phenotype and shift toward an apoptotic state. The degeneration of reparative Schwann cells is a multistage process that begins with a decline in the reparative phenotype, characterized by the progressive downregulation of repair-associated factors, and ultimately culminates in widespread cell death [4]. The modeling results derived from the endogenous network recapitulate this progression, as shown in Figure 6A. The transitional states connecting the DRSC-steady state and the ASC-steady state are hereafter referred to as the DR to A Transition. The expression profiles of these DR to A Transitions (T2, T5, T10, T11, T12, T17, T21, and T27) are visualized in Figure 6C. Overall, these transitions exhibit low expression of dedifferentiation-related factors, with the cell cycle module completely inactive, and generally low levels of inflammatory and signaling pathways. Importantly, the apoptotic factors caspase-3 (Casp3) and caspase-9 (Casp9) are markedly elevated, indicating activation of the apoptotic pathway. This suggests that the corresponding cellular states have begun to undergo programmed cell death, which explains why they are connected to the ASC-steady state. This systems-level analysis extends transcriptome-based observations by showing that loss of repair identity is closely linked to the activation of apoptosis. Together, these results suggest that the DR to A transition follows a structured, rather than stochastic, cell fate trajectory, providing a dynamical framework for understanding how repair-deficient Schwann cells progress toward apoptosis.

Notably, several transitional states (T5, T12, and T21) simultaneously connect the RSC-steady state with the DRSC-steady state, as well as the DRSC-steady state with the ASC-steady state. This topological feature suggests that these states occupy a central position within the regulatory landscape, acting as critical transition hubs rather than simple intermediate states along a linear trajectory. The dual connectivity of these states indicates that repair-deficient Schwann cells are not irreversibly committed to degeneration. Instead, they reside within a dynamically unstable region. From this state, cells may either revert to a reparative phenotype or progress toward apoptosis. This further implies the existence of a potential decision window during which targeted interventions could redirect cell fate, highlighting these transitional states as key control points in the regulation of Schwann cell repair capacity. Together, these findings reveal that the transition from repair to degeneration is not a linear process, but is instead governed by a branched and dynamically regulated state-space architecture.

### 3.5. Prediction of Potential Therapeutic Targets

Based on the above analyses, we further investigated potential targets for restoring the repair capacity of Schwann cells undergoing functional decline. R to DR transitions (T3, T21) exhibit reduced expression of inflammatory modules and attenuation of key signaling pathways. Consistently, R to DR transitions (T4, T5, T12, T 16) display decreased inflammatory activity, suppression of key pathways, downregulation of dedifferentiation-associated factors—including c-Jun, BDNF, and Stat3—and increased activation of apoptotic modules. Collectively, these findings suggest that enhancing inflammatory signaling, reactivating key pathways, upregulating core dedifferentiation factors, and inhibiting apoptosis may represent effective strategies for maintaining the reparative phenotype of Schwann cells. We next leverage the previously constructed network to test these predictions.

In endogenous networks, modulating the expression of specific factors can drive transitions between distinct cellular states. We applied this strategy to validate and simulate targeted interventions in Schwann cells. Our simulated interventions support existing findings while further extending mechanistic insights into Schwann cell (SC) repair capacity. We summarized the effective intervention strategies in Table 3. We first examined the role of c-Jun in Schwann cells. It is well established that c-Jun is a key regulator of Schwann cell dedifferentiation. During chronic denervation, Schwann cells exhibit a downregulation of c-Jun. Researchers established a chronic denervation model in mice by transecting the sciatic nerve. Ten weeks after injury, c-Jun expression was markedly downregulated, leading to impaired Schwann cell repair function and failed nerve regeneration. Maintenance of c-Jun expression was sufficient to rescue this deficit [54]. Similarly, another study established a chronic denervation model in rats and demonstrated that administration of Neurotrophin-3 maintained high levels of c-Jun expression, thereby promoting nerve regeneration [129]. Importantly, loss-of-function experiments further confirmed the central role of c-Jun in Schwann cell-mediated nerve repair. Mice with selective inactivation of c-Jun in Schwann cells exhibited severely impaired regenerative capacity. This demonstrates that c-Jun acts as a global regulator of the Schwann cell injury response. It is essential for specifying the denervated repair Schwann cell state, which encompasses its characteristic gene expression program, cellular structure, and regenerative function [46].

**Figure 7:**
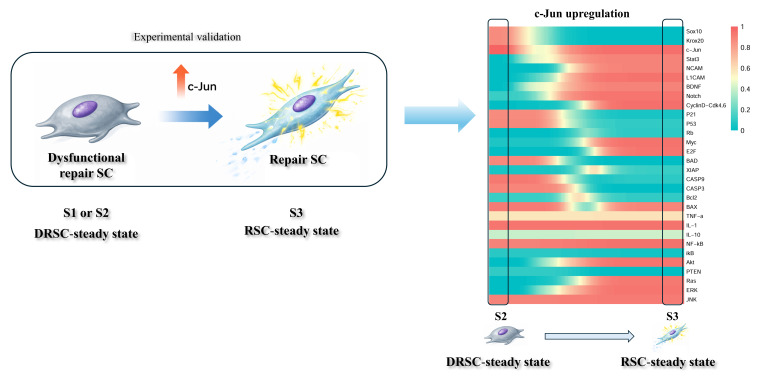
Target predicted by endogenous network modeling for maintaining the reparative capacity of Schwann cells. Upregulation of c-Jun (highlighted in the box) has been experimentally demonstrated to be essential for sustaining the reparative phenotype of Schwann cells [54]. In our model, DRSC-steady state (S2) correspond to dysfunctional repair Schwann cells, whereas RSC-steady state (S3) represents a repair Schwann cell state with robust repair ability. Consistent with experimental observations, enforced upregulation of c-Jun drives the transition from the DRSC-steady state (S2) to the RSC-steady state (S3), thereby restoring the reparative phenotype. The upward red arrow indicates upregulation of c-Jun. Blue arrows in the left panel represent cell state transitions. The blue arrow between the boxed panel and the heatmap denotes agreement between model predictions and experimental validation. The arrow below the heatmap indicates the transition from dysfunctional repair Schwann cell steady state to a repair Schwann cell steady state.

As described above, the DRSC steady state (S2) represents repair-deficient Schwann cells, characterized by reduced c-Jun expression, whereas the RSC steady state (S3) corresponds to normal repair-competent Schwann cells. By simulating upregulation of c-Jun, we were able to drive the DRSC steady state (S2) back to the RSC steady state (S3), see Figure 7. This result indicates that our model recapitulates experimental observations showing that restoring or maintaining c-Jun expression can rescue the regenerative capacity of Schwann cells with impaired repair potential. Together with the genetic loss-of-function evidence, these findings further demonstrate the reliability of our model in capturing the causal role of c-Jun and in predicting effective molecular interventions [175].

**Figure 8:**
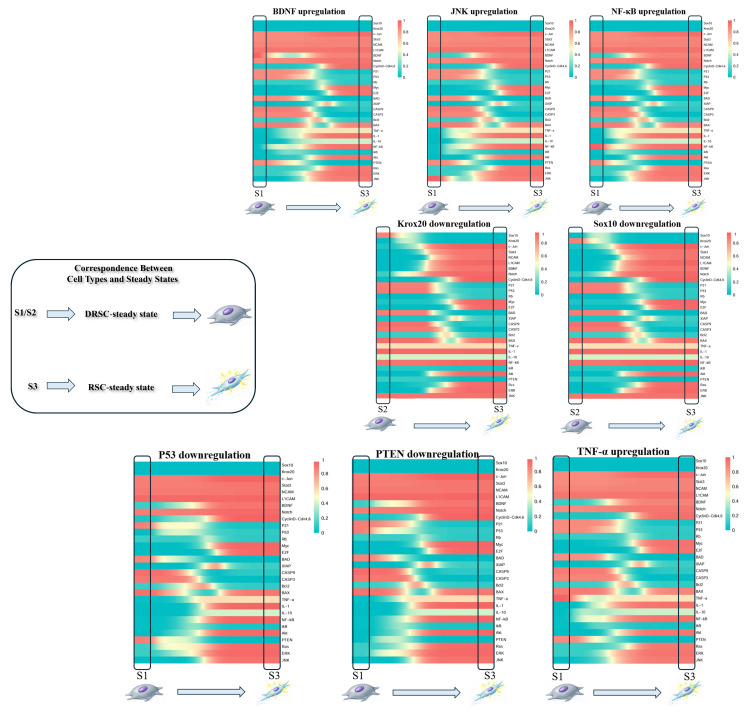
Effects of single-target perturbations on steady state transitions of repair-deficient Schwann cells. Heatmaps show the dynamic changes in gene expression profiles following individual target perturbations, including BDNF upregulation, JNK upregulation, NF-κB upregulation, TNF-α upregulation, as well as downregulation of Krox20, Sox10, P53, and PTEN. Each row represents a gene in the regulatory network, and the color scale (0–1) indicates normalized expression levels (teal: low; red: high). For each perturbation, the system trajectory is recorded from the initial steady state (DRSC-steady state) to the final steady state (RSC-steady state), with intermediate states shown along the horizontal axis. Black boxes highlight the regions corresponding to the initial and final steady states. Upregulation of BDNF, JNK, NF-κB, and TNF-α, as well as downregulation of P53 and PTEN, drives the system from the DRSC-steady state (S1) toward the RSC-steady state (S3). Downregulation of Krox20 and Sox10 induces the transition from the DRSC-steady state (S2) to the RSC-steady state (S3), indicating that appropriate interventions in repair-deficient Schwann cells can maintain their repair capacity. The schematic summary (bottom left) illustrates the correspondence between modeled steady states and biological cell types: S1/S2 correspond to the DRSC-steady state, representing repair-deficient Schwann cells, whereas S3 corresponds to the RSC-steady state, representing normal repair Schwann cells. All arrows in the figure indicate the direction of cell state or steady state transitions.

Researchers investigated the effects of BDNF on the JAK/STAT pathway using four different cell types. The results demonstrated that BDNF can activate the JAK/STAT pathway in both rat and human Schwann cells, and promote the release of the cytokine IL-6 [176]. While the previous study did not specifically examine repair-deficient Schwann cells, these findings nonetheless suggest that BDNF upregulation may serve as a promising intervention strategy. In our simulated intervention experiments, upregulation of BDNF was able to reverse the decline in Schwann cell repair capacity, furthermore, IL-6 was also upregulated in the model (Figure 8). This supplements existing knowledge by suggesting that BDNF is not only a physiological signaling molecule but also a viable therapeutic target capable of restoring impaired regenerative functions in severe or chronic injury contexts.

To our knowledge, there is no direct experimental evidence demonstrating that upregulation of JNK can reverse the decline in Schwann cell repair capacity. Regarding the signaling role of JNK, existing literature presents seemingly contradictory findings. Previous studies using cultured primary Schwann cells have shown that activation of the MKK7–JNK signaling pathway can induce c-Jun expression [177]. However, another study showed that inhibition of JNK in transected nerve explants failed to affect injury-induced c-Jun upregulation, suggesting that c-Jun activation in Schwann cells may, at least in part, occur through JNK-independent mechanisms [178]. Our simulations refine this understanding by demonstrating that while JNK may not be the sole or indispensable initiator of the injury response, its upregulation is sufficient to reverse the decline in SC repair capacity. In our model, upregulation of JNK drives the transition from the DRSC-steady (S1) state to the RSC-steady state (S3). This suggests that JNK may function as a context-dependent modulatory factor, contributing to the restoration, maintenance, and stability of the repair state, rather than acting as an essential upstream driver of c-Jun activation. By distinguishing between these two kinetic phases, the model supplements the existing knowledge, providing a plausible explanation for why JNK appears redundant in acute transection models yet remains a potent target for restoring long-term regenerative potential. This prediction suggests that JNK may contribute to restoring Schwann cell repair capacity when it is impaired, providing a testable hypothesis for future studies.

In addition, our simulated interventions indicate that upregulation of NF-κB and TNF-α is sufficient to maintain Schwann cell repair capacity. While prolonged or excessive inflammation is generally considered detrimental, controlled inflammatory signaling has been shown to support nerve repair [154]. For example, in a rat sciatic nerve injury model, TNF-α was shown to promote Schwann cell activation and macrophage recruitment, thereby facilitating myelin clearance and creating a permissive environment for regeneration [179]. Similarly, in a zebrafish spinal cord injury model, pharmacological suppression of the inflammatory response impaired axonal regeneration, whereas enhancement of inflammation accelerated regrowth, demonstrating that an appropriate level of inflammation is required for effective repair [180].

Moreover, the predicted preservation of repair ability through the downregulation of P53 and PTEN confirms the roles of these molecules as critical molecular brakes during chronic denervation. We speculate that reducing P53 expression may decrease Schwann cells’ sensitivity to chronic denervation, thereby preventing apoptosis or entry into permanent senescence under such conditions, whereas PTEN downregulation likely alleviates inhibition of key pathways such as Akt, promoting Schwann cell survival. While previous studies focused on their roles in axonal regeneration [181], our model extends this understanding by suggesting that their suppression is critical for preventing the decline in Schwann cell repair capacity.

In our simulations, downregulation of Krox20 and Sox10 reversed the phenotype associated with reduced Schwann cell repair capacity. The antagonistic relationship between myelination and repair programs has been well documented [29,47]. Our model builds on this by suggesting that relieving pro-myelinating transcriptional pressure alone may be sufficient to reactivate the repair program. This raises the possibility that the decline in repair capacity during chronic injury is partly driven by the persistent activity of myelination-associated inhibitory signals, providing a testable hypothesis for future studies.

It should be noted that the level of experimental support varies among the predicted intervention targets. The restorative effects of c-Jun are strongly supported by previous experimental studies, while the potential role of BDNF is partially supported by evidence demonstrating its ability to activate repair-associated signaling pathways in Schwann cells. In contrast, the predicted benefits of modulating JNK, NF-κB, TNF-α, P53, PTEN, Krox20, and Sox10 arise primarily from the network dynamics identified by the present model and should therefore be regarded as testable hypotheses requiring further experimental validation. Consequently, these findings are intended to guide future investigation rather than to establish definitive therapeutic strategies.

In summary, our simulations indicate that modulation of key regulatory nodes, including c-Jun, BDNF, JNK, NF-κB, TNF-α, P53, PTEN, Krox20, and Sox10, may help restore or maintain Schwann cell repair capacity. While some predictions, particularly those involving c-Jun and BDNF, are supported by existing experimental observations, several others remain model-derived hypotheses that require further experimental investigation. Collectively, these findings highlight the value of the model in identifying potential molecular targets and guiding future experimental studies aimed at preserving or restoring Schwann cell regenerative capacity.

## 4. Discussion

### 4.1. Biological Significance and Novel Insights from the Dynamical Landscape

In this study, we employed a bottom-up strategy to construct a core endogenous regulatory network of Schwann cells. By formulating dynamical equations, we derived the corresponding potential landscape and systematically characterized the global dynamics of Schwann cell fate regulation. Because no experimental data were used as direct inputs, the resulting landscape is not constrained by data quality or sample size, representing a fundamental distinction from data-driven approaches. This knowledge-based topological foundation captures the major steady and transitional states of Schwann cells in qualitative agreement with existing experimental datasets, allowing us to elucidate the intrinsic mechanisms of cellular plasticity. Beyond mechanistic insights, our model provides a powerful in silico platform to screen therapeutic targets for peripheral nerve injury. By simulating genetic perturbations, our model successfully recapitulated experimental observations—such as the rescue of declining repair capacity in chronic denervation via c-Jun upregulation [54]. Furthermore, we identified a broader spectrum of potent interventional targets, including BDNF, JNK, NF-κB, TNF-α, p53, PTEN, Krox20, and Sox10, which can similarly drive the transition of functionally declining Schwann cells back into a normal reparative state. In future studies, we will systematically explore combinations of these genetic perturbations to devise optimal therapeutic strategies.

Overall, our model provides new biological insights that go beyond the known transcriptional networks. First, we established a quantitative framework for Schwann cell fate regulation based on complex systems theory. This study overcomes the limitations of traditional approaches that focus on single molecules and linear signaling pathways by integrating complex systems theory with nerve repair research, thereby characterizing the dynamic process of Schwann cell fate transitions at the systems level. By introducing nonlinear dynamical modeling, we construct for the first time a multi-layered network—from functional modules to signaling pathways to key factors—that captures the interactions among regulatory elements and their collective behavior, providing a novel theoretical framework for understanding Schwann cell phenotypic transitions. Furthermore, our model improves the understanding of repair Schwann cell decline during chronic injury by revealing both the dynamic transition trajectories leading to repair failure and the underlying mechanisms, including the emergence of apoptosis-prone subpopulations and distinct pathways driving the progression toward apoptosis.

Importantly, for the first time, this study computationally derived the topological potential landscape of Schwann cell dedifferentiation, systematically recapitulating the dynamic trajectories from the normal state to the reparative state and further revealing multiple potential paths leading from declining repair capacity to apoptosis. These results not only overcome the limitations of experimental studies, which cannot continuously track cellular state transitions, but also provide a novel perspective for understanding the intrinsic dynamical mechanisms underlying Schwann cell fate differentiation.

Furthermore, we constructed a dynamical model that does not rely on high-throughput data yet achieves effective correspondence with experimental observations. Unlike studies that depend on large-scale omics data for correlation-based analyses, our model does not require external high-throughput data as input. Instead, it is built upon existing biological knowledge to establish the regulatory network, and steady states are obtained through nonlinear dynamical computations. The expression features of these predicted steady states correspond well with both low-throughput experimental results and transcriptomic data, demonstrating the potential of this approach for mechanistic interpretation and theoretical prediction even in the absence of large-scale datasets.

Finally, we predicted that dedifferentiated reparative Schwann cells may exhibit intrinsic heterogeneity based on differences in molecular expression across transitional states. In our simulations, the transitional state T16 connecting the myelinating and reparative steady states showed higher expression of apoptotic factors, potentially representing a Schwann cell subpopulation that is more prone to apoptosis; thus, compared with typical reparative Schwann cells, these cells are expected to have shorter lifespans. Experimental studies indicate that, following delayed nerve repair, the number of Schwann cells isolated from distal nerve stumps gradually decreases, suggesting that reparative Schwann cells are intrinsically heterogeneous: subpopulations with distinct stability and apoptosis sensitivity may undergo selective cell death at different stages, leading to the observed decline in cell numbers [163,171]. However, to date, no studies have specifically targeted these apoptosis-prone subpopulations. Identification and targeted modulation of these apoptosis-prone subpopulations may provide new opportunities for therapeutic intervention in peripheral nerve injury.

In general, building on the currently established core endogenous network of Schwann cell dedifferentiation, we constructed a topological landscape to describe the dedifferentiation process. However, beyond capturing key processes such as inflammation, differentiation, proliferation, and apoptosis in adult Schwann cells following injury, we have not yet addressed fate determination during embryonic development and organismal aging, such as the differentiation of Schwann cell precursors and immature Schwann cells [1]. Therefore, our computational results are currently limited to describing Schwann cell dedifferentiation after nerve injury. Schwann cell phenotypic plasticity is not only critical for nerve repair but also potentially contributes to regeneration in other tissues [182]. Future work could incorporate key modules and factors related to embryonic Schwann cell development and aging to expand the endogenous network, thereby linking developmental processes, post-injury cellular behaviors, and age-associated functional decline. This approach would enable the analysis of differences in their underlying dynamical mechanisms, providing new insights for precise modulation of Schwann cell fate and offering a theoretical foundation for regenerative medicine and clinical research.

### 4.2. Limitations of the Study

Several aspects of the present framework should be considered when interpreting the model predictions. First, the endogenous regulatory network was constructed using 30 key factors that have been widely implicated in Schwann-cell biology and peripheral nerve regeneration. The objective of the model was not to include every molecule involved in nerve injury and repair, but rather to capture the core regulatory architecture underlying Schwann-cell fate transitions. Consequently, certain potentially relevant regulatory systems were not incorporated into the current framework. For example, signaling pathways such as the NOS/NO system have been reported to interact with several components represented in the network [183,184,185]. However, because the biological effects of NO are highly concentration-dependent and vary across different cellular contexts, making its regulatory topology difficult to define consistently, the NOS/NO system was not explicitly incorporated in order to preserve network robustness and interpretability. As additional biological evidence becomes available, the framework can be further expanded by incorporating additional regulatory factors and molecular processes.

Second, the represented regulatory interactions constitute a coarse-grained description of the endogenous regulatory system. To enable the analysis of global network dynamics and attractor-state transitions, experimentally reported regulatory relationships were abstracted into a dynamical framework describing the principal modes of interaction among genes, transcription factors, and signaling pathways. Consequently, the model is designed to reveal the overall regulatory landscape, dominant transition pathways, and key control nodes associated with Schwann-cell fate determination, rather than to reproduce every molecular event occurring in vivo. Therefore, the predictions generated by the model should be interpreted primarily at the systems level, including the identification of candidate intervention targets, regulatory pathways, and cell-state transition mechanisms. Future studies may further enhance the biological detail of the framework by incorporating additional molecular components and regulatory processes.

Third, model validation was performed using independent low-throughput experiments, bulk transcriptomic datasets, and single-cell RNA sequencing data. These complementary approaches provide validation at the molecular, transcriptomic, and cell-state levels, respectively. Together, they support the biological relevance of the predicted steady states and transition trajectories. As additional experimental datasets become available, the framework can be further evaluated across a broader range of biological conditions and experimental settings.

Finally, the present model primarily captures regulatory interactions at the transcriptional and signaling levels, whereas post-translational regulatory mechanisms were not explicitly represented. In particular, processes such as phosphorylation, ubiquitination, and S-nitrosylation can rapidly modulate protein activity, stability, and signaling output without corresponding changes in gene expression. As a result, these regulatory layers may introduce fast and transient dynamics that are not reflected in the current framework, potentially leading to deviations between model-predicted states and short-term cellular responses. Therefore, the predictive results of the model should be interpreted as representing the dominant and relatively stable regulatory tendencies of the endogenous network, rather than fully quantitative descriptions of all molecular events. Despite this limitation, the core transcriptional regulatory architecture of Schwann-cell fate regulation is preserved in the model. The framework thus remains useful for identifying key regulatory nodes, characterizing phenotype transitions, and generating testable hypotheses. In particular, it provides a systems-level platform for analyzing how perturbations propagate through the regulatory network and for prioritizing candidate intervention targets. By systematically evaluating single and combinatorial perturbations, the model may facilitate the design of future experimental studies in peripheral nerve regeneration and regenerative medicine research.

## 5. Conclusions

In this study, we constructed an endogenous regulatory network of Schwann cells and investigated its dynamical behavior using systems-level modeling and landscape analysis. The model identified multiple stable cellular states corresponding to myelinating, non-myelinating, repair, dysfunctional repair, and apoptotic Schwann cell phenotypes. By analyzing the transition pathways among these states, we revealed a progressive trajectory through which repair Schwann cells gradually lose regenerative competence during chronic denervation and ultimately undergo apoptosis. In particular, the predicted heterogeneity among repair Schwann cells provides a potential mechanistic explanation for the staged apoptotic loss of Schwann cells observed experimentally. These findings further suggest that repair Schwann cell subpopulations with increased apoptotic susceptibility may constitute a previously underappreciated therapeutic target for promoting nerve regeneration.

More broadly, the present work supports the view that cellular state transitions during tissue repair emerge from the collective behavior of endogenous regulatory networks. By linking molecular regulation to phenotypic dynamics within a unified framework, this study offers new insight into Schwann cell fate control and provides a foundation for the rational identification of intervention targets aimed at preserving regenerative competence following peripheral nerve injury.

## Figures and Tables

**Table 1:** Preliminary categorization of steady states derived from the endogenous regulatory network of Schwann cells. Group 1, RSC, repair Schwann cells; Group 2, myelinating Schwann cell (MSC), myelinating Schwann cells; Group 3 NMSC, non-myelinating Schwann cells; Group 4, Death, cells with apoptotic propensity or undergoing cell death.

	Group 1		Group 2				Group 3			Group 4		
Steady state	S1	S3	S2	S4	S6	S7	S9	S10	S11	S5	S8	S12
Differentiation	RSC		MSC				NMSC			Death		
Proliferation	off	on	off	off	off	off	off	off	off	off	off	off
Apoptosis	on	off	on	on	off	on	on	off	on	on	on	off
Inflammation	off	on	on	off	off	off	off	off	off	off	off	off
Pathway	off	on	off	off	off	off	off	off	off	off	off	off

**Table 2:** The left panel summarizes, based on experimental evidence, the distinct expression states of myelinating, non-myelinating, and repair Schwann cells across key functional modules. The right panel presents three representative steady states (S6, S10, and S3) derived from the endogenous network model, which are preliminarily classified as myelinating, non-myelinating, and repair Schwann cells, respectively, based on differentiation module markers.

Experimental Knowledge				Model Steady State		
Cell type	MSC	NMSC	RSC	S6	S10	S3
Proliferation	off	off	on	off	off	on
Apoptosis	off	off	off	off	off	off
Inflammation	off	off	on	off	off	on
Pathway	off	off	on	off	off	on

**Table 3:** For the two Schwann cell states with dysfunctional repair capacity (S1 and S2), we systematically upregulated or downregulated each regulatory factor within these steady states to assess whether they could be reprogrammed into an active, pro-repair phenotype. The resulting transitions are summarized in the table, which highlights the specific intervention strategies capable of driving these dysfunctional states toward a repair Schwann cell (RSC) identity.

Steady State	Interference Target	Interference Measures	Result	Cell Type	Evidence Type
S1 DRSC-steady state	BDNF	Downregulation	S1	Dysfunctional RSC	\
	BDNF	Upregulation	RSC-steady state	RSC	Experimentally supported
	JNK	Downregulation	S1	Dysfunctional RSC	\
	JNK	Upregulation	RSC-steady state	RSC	Prediction only
	NF-κB	Downregulation	S1	Dysfunctional RSC	\
	NF-κB	Upregulation	RSC-steady state	RSC	Prediction only
	TNF	Downregulation	S1	Dysfunctional RSC	\
	TNF	Upregulation	RSC-steady state	RSC	Prediction only
	P53	Downregulation	RSC-steady state	RSC	Prediction only
	P53	Upregulation	S1	Dysfunctional RSC	\
	PTEN	Downregulation	RSC-steady state	RSC	Prediction only
	PTEN	Upregulation	S1	Dysfunctional RSC	\
S2 DRSC-steady state	c-Jun	Downregulation	S2	Dysfunctional RSC	\
	c-Jun	Upregulation	RSC-steady state	RSC	Experimentally supported
	Krox20	Downregulation	RSC-steady state	RSC	Prediction only
	Krox20	Upregulation	S2	Dysfunctional RSC	\
	Sox10	Downregulation	RSC-steady state	RSC	Prediction only
	Sox10	Upregulation	S2	Dysfunctional RSC	\

## Data Availability

The data that support the findings of this study are available from the corresponding author upon reasonable request.

## Supplementary Materials

The following supporting information can be downloaded at: https://www.mdpi.com/article/10.3390/biology15131079/s1, Table S1: Modules and factors of endogenous network in Schwann cells; Table S2: Regulatory relationships among factors in the network; the regulatory network was constructed based on the references listed in [31,43,50,55,56,57,58,59,60,61,62,63,64,65,66,67,68,69,70,71,72,73,74,75,76,77,78,79,80,81,82,83,84,85,86,87,88,89,90,91,92,93,94,95,96,97,98,99,100,101,102,103,104,105,106,107,108,109,110,111,112,113,114,115,116,117,118,119,120,121,122,123,124,125,126,127,128,129,130,131,132,133,134]. Table S3: 12 stable states derived by differential equation method and gene expression levels; Table S4: 47 transition states derived via the differential equation method; Table S5: 37 attractors calculated by the Boolean algebra method. Excel File: supplemental convergence analysis.

## Abbreviation

The following abbreviations are used in this manuscript:

Sox10	SRY (sex determining region Y)-box transcription factor 10
Krox20	Early growth response 2 (EGR2)
c-Jun	Jun proto-oncogene, AP-1 transcription factor subunit
Stat3	Signal transducer and activator of transcription 3
NCAM	Neural cell adhesion molecule 1
L1CAM	L1 cell adhesion molecule
BDNF	Brain derived neurotrophic factor
Notch	Notch receptor 1
CyclinD-Cdk4,6	Cyclin D–cyclin-dependent kinase 4/6 complex
P21	Cyclin dependent kinase inhibitor 1A
P53	Tumor protein p53
Rb	RB transcriptional corepressor 1
Myc	MYC proto-oncogene, bHLH transcription factor
E2F	E2F transcription factor
BAD	BCL2 associated agonist of cell death
XIAP	X-linked inhibitor of apoptosis
CASP9	Caspase 9
CASP3	Caspase 3
Bcl2	BCL2 apoptosis regulator
BAX	BCL2-associated X protein
TNF-α	Tumor necrosis factor
IL-1	Interleukin 1 beta
IL-10	Interleukin 10
NF-κB	Nuclear factor kappa B
iκB	Inhibitor of nuclear factor kappa B
Akt	AKT serine/threonine protein kinase
PTEN	Phosphatase and tensin homolog
Ras	Ras signaling pathway
ERK	Extracellular signal-regulated kinase
JNK	c-Jun N-terminal kinase
ODE	Ordinary differential equation
DRSC	Declining repair Schwann cell
ASC	Apoptotic Schwann cell
RSC	Repair Schwann cell
MSC	Myelinating Schwann cell
NMSC	Non-myelinating Schwann cell
